# The Role of Adenosine A2b Receptor in Mediating the Cardioprotection of Electroacupuncture Pretreatment via Influencing Ca^2+^ Key Regulators

**DOI:** 10.1155/2019/6721286

**Published:** 2019-12-02

**Authors:** Qiu-Fu Dai, Jun-Hong Gao, Juan-Juan Xin, Qun Liu, Xiang-Hong Jing, Xiao-Chun Yu

**Affiliations:** ^1^Acupuncture and Moxibustion Department, Beijing Hospital of Traditional Chinese Medicine Affiliated to Capital Medical University, Capital Medical University, Beijing Key Laboratory of Acupuncture Neuromodulation, 23 Art Museum Back Street, Beijing 100700, China; ^2^Institute of Acupuncture and Moxibustion, China Academy of Chinese Medical Sciences, 16 Nanxiaojie, Dongzhimennei, Beijing 100700, China

## Abstract

**Objective:**

To investigate the roles played by A2b receptor and the key Ca^2+^ signaling components in the mediation of the cardioprotection of electroacupuncture pretreatment in the rats subjected to myocardial ischemia and reperfusion.

**Methods:**

SD rats were randomly divided into a normal control (NC) group, ischemia/reperfusion model (M) group, electroacupuncture pretreatment (EA) group, and electroacupuncture pretreatment plus A2b antagonist (EAG) group. The ischemia/reperfusion model was made by ligation and loosening of the left descending branch of the coronary artery in all groups except the NC group. The EA group was pretreated with electroacupuncture at the *Neiguan* (PC6) point once a day for three consecutive days before the modeling. The elevation of the ST segment, arrhythmia scores, and myocardial infarction size of each group was measured. The relative expression levels of A2b, RyR2, SERCA2a, NCX1, P-PLB(S16)/PLB, and Troponin C/Troponin I proteins in the injured myocardium were detected by multiple fluorescence western blot.

**Results:**

The level of ST segment, arrhythmia scores, and infarct size in the M group was significantly higher/larger than that in the NC group after ischemia and reperfusion, while all the three indices mentioned above in the EA group were significantly lower/smaller than those in the M group after reperfusion. The expression of the proteins of adenosine receptor 2b(A2b), ryanodine receptor 2(RyR2), and sarco(endo)plasmic reticulum Ca^2+^-ATPase 2a (SERCA2a) in the EA group was significantly enhanced as compared with the M group, while in the EAG group, the contents of A2b were significantly lower than those in the EA group, and RyR2 was higher in the EAG group. In comparison with the NC group, the relative expression of NCX1 protein in M, EA, and EAG groups was not changed significantly. The ratio of phosphorylated phospholamban (P-PLB) over phospholamban (PLB) in the M group was significantly lower than that in the NC group, and the ratio in the EA group was significantly increased as compared with the M group, while the ratio of Troponin C/Troponin I in the EA group was significantly decreased in comparison with that in other groups.

**Conclusion:**

Electroacupuncture pretreatment could reduce ischemia and reperfusion-induced myocardial injury via possibly increasing the A2b content and regulating the key Ca^2+^ signaling components, namely inhibiting RyR2 and enhancing P-PLB(S16)/PLB ratio and SERCA2a proteins, so as to diminish the intracellular Ca^2+^ overload and consequently lessen the myocardial injury.

## 1. Introduction

Ischemic heart disease (IHD) is one of the diseases with the highest morbidity and mortality over the world. In China, there were about 4 million patients attacked by the coronary heart disease in 2016 [1]. The studies showed that in patients with IHD, a further myocardial injury can be caused by the ischemia/reperfusion(I/R) [2, 3]. In the recent decades, it has been a hot topic to find out a safe and effective approach to the prevention and treatment of the reperfusion-induced myocardial injury. Ely and his colleagues reported [4] previously that adenosine released during myocardial ischemia produced a direct cardioprotection. Adenosine receptors were reported to mediate not only the cardioprotection induced by ischemic preconditioning [5, 6] but also the inhibition of the apoptosis of cardiac cells during the reperfusion [7]. Among the well-known 4 adenosine receptors, the subtype adenosine receptor 2b (A2b) was proved to mediate the cardioprotective effects induced by both ischemic preconditioning and postconditioning [8]. In the rats with A2b gene knocked out, there were not any cardioprotective effects observed, while the ischemic preconditioning could still produce the cardioprotection in rats with A1, A2a, or A3 gene knocked out [9]. The results indicated that A2b receptor played an important role in the mediation of the cardioprotection. It was showed in the clinical studies that the myocardial injury was reduced effectively by the acupuncture pretreatment in patients with myocardial ischemia [10, 11]. The results achieved by a lot of experimental studies indicated that the incidence rates of sudden death [12], arrhythmias, and angina pectoris were significantly diminished by acupuncture [13, 14]. Acupuncture stimulation was also showed to alter both the local adenosine concentration in the tissues around the acupoints [15] and the expression of A2b receptor in cardiac cells [16]. Accordingly, it is highly likely that A2b participates in the cardioprotection produced by acupuncture pretreatment. It is well known that intracellular calcium overload contributes to the myocardial ischemic injury, and A2b is involved in the modulation of the intracellular calcium concentration [17–19]. The aim of present study is to investigate the role played by A2b receptor and the key Ca^2+^ signaling components in the mediation of the cardioprotection produced by acupuncture pretreatment. The outcomes will provide the scientific evidence to support acupuncture as an applicable way to effectively prevent and control the IHD.

## 2. Materials and Methods

### 2.1. Animals

Forty-eight male Sprague Dawley (SD) rats weighing 300 ± 25 g were purchased from Experimental Animal Central of Peking Union Medical University (Certifcate number SCX2016-0002, Beijing, China). The rats were kept in an animal house maintained at 21 ± 2°C with a 12-hour light-dark cycle and freed to have food and water. All experiments conducted in the present studies involving animals were in accordance with the ethical standards of the Institutional Animal Care and Use Committee of the China Academy of Chinese Medical Sciences.

### 2.2. Grouping and Pretreatment

**Figure 1:**
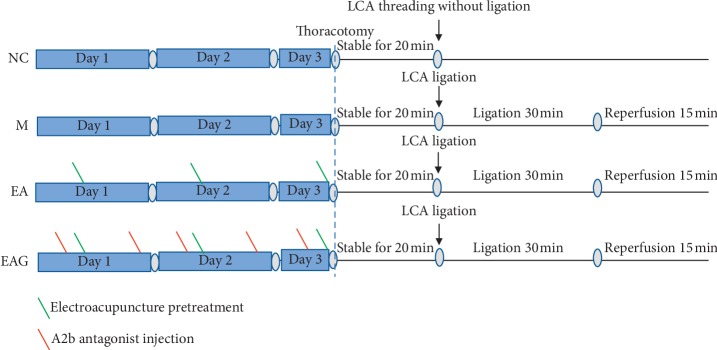
The experimental protocol.

The rats were acclimatized for a week, and randomized into a Normal control (NC) group, Model (M) group, Electroacupuncture pretreatment (EA) group, and Electroacupuncture pretreatment plus A2b antagonist (EAG) group, with 12 rats in each group. The experimental protocol was described in Figure 1. The NC group was only punctured under the left descending branch of coronary artery (LCA) without ligation and electroacupuncture pretreatment. The root of LCA was ligated in the M group without electroacupuncture pretreatment, and the rats in the NC group received threading but not ligation. The EA group was pretreated with electroacupuncture pretreatment applied at bilateral *Neiguan* (PC6) acupoints for 30 min once a day for 3 consecutive days. The acupuncture needle was 0.3 × 25 mm (Huatuo, China), and needling depth was about 2 mm. An A2b antagonist GS6201 (Tocris Bioscience, No.4727) was administered intraperitoneally in the EAG group at a dose of 1 mg/kg, 2 h before EA pretreatment twice a day for 3 consecutive days [20]. The acupoints were located in the forelimbs according to the textbook of experimental acupuncture in animals and stimulated with an intensity of 1 mA and a frequency of 2/10 Hz in the present study.

### 2.3. Acute Myocardial Ischemia and Reperfusion Model

The rats were anesthetized by intraperitoneal injection of 20% urethane (0.5 ml/100 g) and placed on a temperature-controlled heated table to maintain body temperature at 37°C. The trachea was intubated for artificial respiration. After a recovery of 20 min following the thoracotomy, LCA was ligated for 30 min, and the model was established according to our previous research [21]. Successful LCA occlusion was confirmed by an immediate color change of the myocardium below the ligation site from red to dark violet, as well as the immediate occurrence of ST elevations in the electrocardiogram (ECG). After 30-min ischemia, the rats received reperfusion for 15 min among the M, EA, and EAG groups.

### 2.4. Observation and Detection Methods

1. ST segment changes in ECG: the ST segment is defined as 13 ms after the S wave [22]. The ST segment elevation was measured before coronary ligation and 15 min and 30 min after ligation, as well as 15 min after reperfusion, respectively.
2. Arrhythmia scoring system: Curtis and Walker (1988) arrhythmia scoring method was used [23, 24]; the arrhythmias of each group were scored within 15 min after reperfusion, and the details of the scoring system are as follows (Table 1).
3. Determination of ischemic risk zone and infarct size.

The ischemic risk zone and infarct size were determined by Evans blue-TTC double staining. Experimental methods refer to reference [25]; the precooling saline was used to wash off redundant dye from the removed heart, excess water was blotted up by using a filter paper, and the heart was frozen to −20°C for 15 min. The frozen myocardium was cut into 5 thin slices with a thickness of 2 mm along the ligation position. The digital camera was used to take pictures. Image-Pro plus 6.0 software was used to calculate the infarct size and risk zone. After staining, the myocardial tissue showed blue as normal tissue, red as risk zone, and pale as infarct size. The ratio of infarct size/risk zone represents the extent of the infarct.

### 2.5. Multiple Fluorescence Western Blot

Cardiac tissue was cracked by RIPA and phosphatase inhibitor (Bimake, B15001) mixture to extract protein. Protein concentration was determined by BCA protein reagents (Thermo scientific, no. 23227). 20 *μ*g of proteins were resolved on SDS-PAGE, transferred to a low-fluorescence PVDF membrane (Thermo scientific, no. 22860), and incubated with the following primary antibodies: anti-A2b (1 : 1000, GeneTex54903), antiphospholamban (PLB, 1 : 5000, Abcam2865), antiphospholamban phosphor S16 (P-PLB, 0.5 *μ*g/ml, Abcam15000), antiryanodine receptor 2 (RyR 2, 1 : 5000, Abcam2861), NCX1 (1 : 1000, Abcam177952), SERCA2a (1 : 1000, Abcam2861), troponin C (1 : 4000, Abcam137130), and troponin I (1 : 2000, Abcam10231). All of the membranes were incubated at 4°C overnight. After incubation with fluorescent secondary antibodies (1 : 15000, Licor, IRDye® 800CW, IRDye® 680RD) for 1 h at room temperature, the membrane was washed 3 times and incubated with the housekeeping protein HFAB™ Rhodamine anti-GAPDH (1 : 1000, Bio-rad 12004167) for 1 h at room temperature. Images were taken with a Typhoon FLA9500 (GE Healthcare) and analyzed by using ImageQuant TL software.

### 2.6. Statistical Analysis

All data are expressed as mean ± SEM. SPSS13.0 software was used for statistical analysis. Normality and equality of variance were tested by the Shapiro–Wilk and Levene test, respectively. Significant difference between groups was determined by one-way ANOVA followed by the Bonferroni *post hoc* test. Arrhythmia scoring was tested by the Kruskal–Wallis H test. *P* < 0.05 was considered as statistically significant.

## 3. Results

### 3.1. The Effects of Electroacupuncture Pretreatment on ECG ST Segment

**Figure 2:**
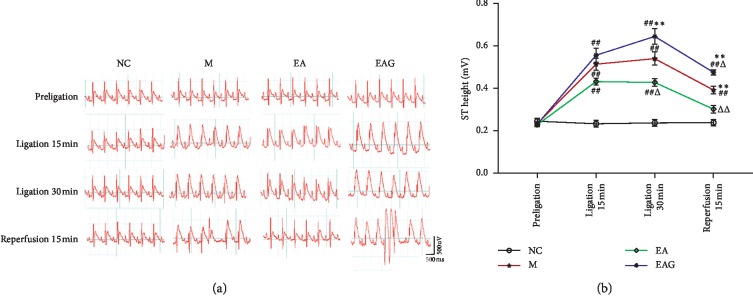
The ECG and ST segments in different groups. (NC = normal control group, M = model group, EA = electroacupuncture pretreatment group, and EAG = electroacupuncture pretreatment plus A2b antagonist group. ^##^*P* < 0.01, ^#^*P* < 0.05, vs. NC group; ^ΔΔ^*P* < 0.01, ^Δ^*P* < 0.05, vs. M group; ^*∗∗*^*P* < 0.01, ^*∗*^*P* < 0.05, vs. EA group, *n* = 12 in each group, $\bar{\chi }\pm SE$).

There was no statistically significant difference in the ST segment among the NC, M, EA, and EAG group before ligation (*P* > 0.05, respectively, Figure 2). The ST segment in the M, EA, and EAG group was significantly increased after ligation and reperfusion as compared with that in the NC group (*P* < 0.01 respectively). The results indicate that the abnormal elevation of the ST segment of ECG was caused by myocardial ischemia and reperfusion. However, the elevation of the ST segment in the EA group was significantly lower than that in the M group at the same time points as mentioned above during ligation for 30 min and reperfusion (*P* < 0.05, *P* < 0.01). The results show that electroacupuncture pretreatment can significantly inhibit the abnormal elevation of the ST segment caused by myocardial I/R injury. Interestingly, the elevation of the ST segment in the EAG group was obviously higher than that in the EA group, indicating that the inhibitory effect of EA pretreatment on the elevated ST segment was reversed by the pretreatment with the specific A2b antagonist.

### 3.2. Arrhythmia Scoring of Different Groups

**Figure 3:**
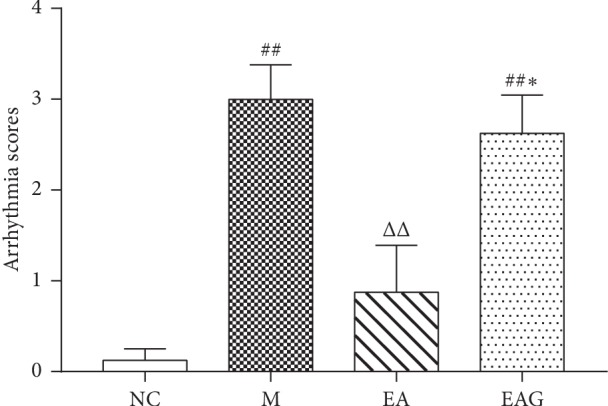
Arrhythmia scoring of different groups. (^##^*P* < 0.01, ^#^*P* < 0.05, vs. NC group; ^ΔΔ^*P* < 0.01, ^Δ^*P* < 0.05, vs. M group; ^*∗∗*^*P* < 0.01, ^*∗*^*P* < 0.05, vs. EA group, *n* = 8 in each group, $\bar{\chi }\pm SE$).

As compared with the NC group, arrhythmia scores in the M group was significantly increased (all *P* < 0.01, Figure 3), while the score in the EA group was significantly decreased (*P* < 0.01). In the EAG group, the score was significantly increased as compared with the EA group (*P* < 0.05).

### 3.3. Risk Zone/Infarct Size in Different Groups

**Figure 4:**
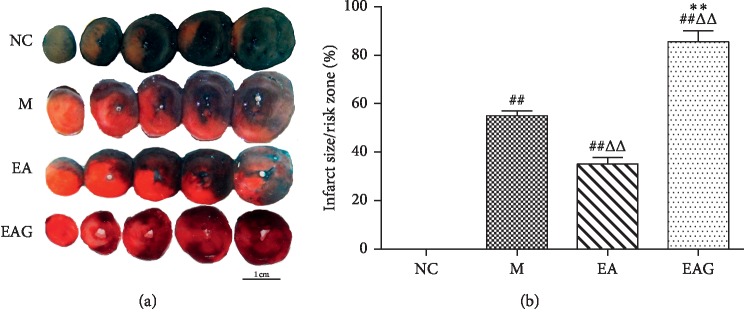
The typical photos of Evans blue-TTC double staining (a) and the statistical graph showing the myocardial infarct size as percentage of the risk zone in the different groups (b). (^##^*P* < 0.01, ^#^*P* < 0.05, vs. NC group; ^ΔΔ^*P* < 0.01, ^Δ^*P* < 0.05, vs. M group; ^*∗∗*^*P* < 0.01, ^*∗*^*P* < 0.05, vs. EA group, *n* = 4 in each group, $\bar{\chi }\pm SE$).

In comparison with the NC group, the infarct size in the M group was obvious, showing that the myocardial injury was caused successfully by ischemia and reperfusion (Figure 4). The infarct size in the EA group was significantly smaller than that in the M group, which indicates that electroacupuncture pretreatment could reduce the myocardial infarction caused by myocardial ischemia. The ratio of infarct size/risk zone in the EAG group was significantly higher than that in the EA group, suggesting that the A2b antagonist (GS6201) can reduce the the cardioprotective effect of electroacupuncture pretreatment.

### 3.4. The Relative Expression of the Proteins of A2b, RyR2, NCX1, and SERCA2a in the Myocardium

**Figure 5:**
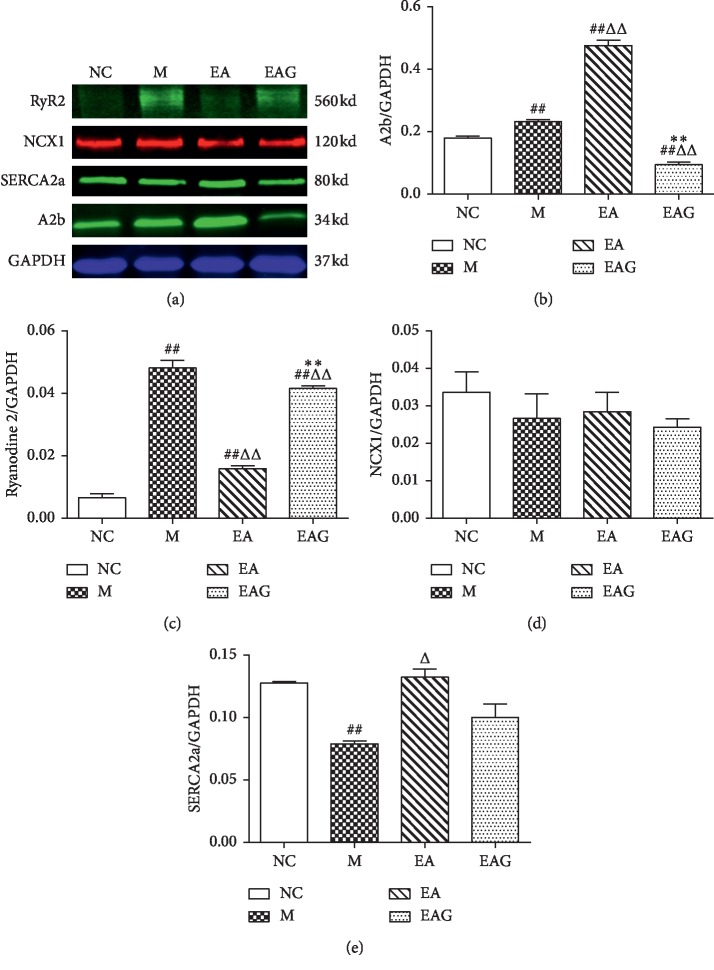
The relative expression of protein in local tissue of injured myocardial tissues. (^##^*P* < 0.01, ^#^*P* < 0.05, vs. NC group; ^ΔΔ^*P* < 0.01, ^Δ^*P* < 0.05, vs. M group; ^*∗∗*^*P* < 0.01, ^*∗*^*P* < 0.05, vs. EA group, *n* = 6 in each group, $\bar{\chi }\pm SE$).

The relative expression of A2b in the M group was significantly higher than that in the NC group (*P* < 0.01). As compared with the M group, A2b content in the EA group was much higher (*P* < 0.01). In the EAG group, A2b content was significantly lower in comparison with EA group, even lower than that in NC group (*P* < 0.01) (Figure 5(b)). The relative expression of RyR2 protein in the M group was much higher than that in the NC group. However, as compared with the M group, the content of RyR2 in the EA group was significantly lower (*P* < 0.01). In the EAG group, the content of RyR2 protein was significantly higher than that in the EA group (*P* < 0.01) (Figure 5(c)). As compared with the NC group, the relative expression of NCX1 in the M, EA, and EAG group did not change significantly (*P* > 0.05) (Figure 5(d)). SERCA2a content in the M group was significantly lower than that in the NC group (*P* < 0.01), while as compared with the M group, the protein content in the EA group was significantly higher (*P* < 0.05). In the EAG group, it was reduced a little bit but not significantly in comparison with the EA group (Figure 5(e)).

### 3.5. The Ratio of P-PLB(S16)/PLB and Troponin C/Troponin I

**Figure 6:**
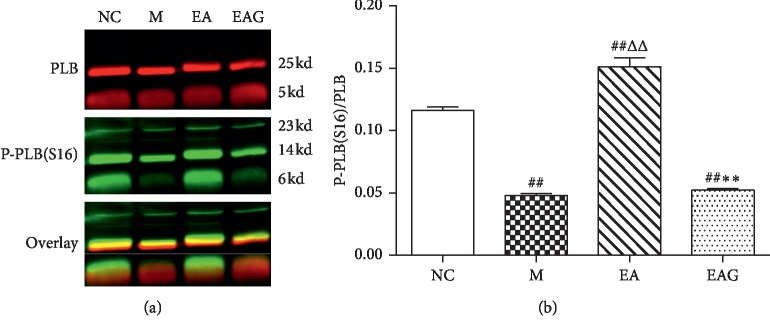
The ratio of P-PLB/PLB in local tissue of injured myocardial tissues. (^##^*P* < 0.01, ^#^*P* < 0.05, vs. NC group; ^ΔΔ^*P* < 0.01, ^Δ^*P* < 0.05, vs. M group; ^*∗∗*^*P* < 0.01, ^*∗*^*P* < 0.05, vs. EA group, *n* = 6 in each group, $\bar{\chi }\pm SE$).

The P-PLB/PLB ratio of the M and EAG group was significantly lower than that of the NC group (*P* < 0.01), while the ratio of the EA group was higher than that of the NC and M group (*P* < 0.01). The ratio of the EAG group was significantly lower than that of the EA group (*P* < 0.01) (Figure 6).

**Figure 7:**
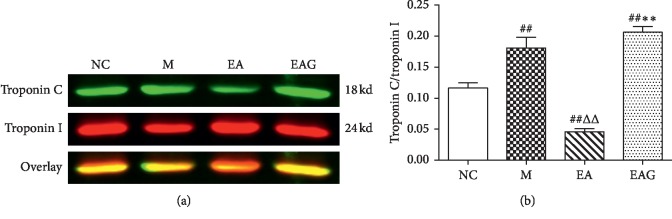
The ratio of troponin C/troponin I in local tissue of injured myocardial tissues. (^##^*P* < 0.01, ^#^*P* < 0.05, vs. NC group; ^ΔΔ^*P* < 0.01, ^Δ^*P* < 0.05, vs. M group; ^*∗∗*^*P* < 0.01, ^*∗*^*P* < 0.05, vs. EA group, *n* = 6 in each group, $\bar{\chi }\pm SE$).

The troponin C/troponin I ratio of the M and EAG group was significantly higher than that of the NC group (*P* < 0.01), while the ratio of the EA group was significantly lower than that of the NC and M group (*P* < 0.01). The ratio of the EAG group was significantly lower than that of the EA group (*P* < 0.01) (Figure 7).

## 4. Discussion

The interesting findings in the present study were that the acupuncture pretreatment could reduce the elevated ECG ST segments, cardiac arrhythmias, and myocardial infarct size significantly in the rats subjected to the myocardial ischemia and reperfusion via enhancing the content of A2b receptor and regulating the expression of the key calcium signaling components including RyR2, SERCA2a, and P-PLB(S16)/PLB and subsequently impact the ratio of Troponin C/Troponin I in cardiac muscle (Table 2).

Previous studies have shown that EA pretreatment could prevent myocardial infarction injury by regulating the AMPK/PGC-1α signaling pathway and AMPK-dependent autophagy process [26, 27], and more and more researchers concern about the acupuncture effects on adenosine receptors. A2b is known to be the major adenosine receptor contributing to the cardioprotection induced by both ischemic preconditioning and postconditioning [28]. Another way for it to mediate the reduction of the ischemia-reperfusion-induced myocardial injury is that activation of the receptor could directly relax the blood vessel and facilitate the regeneration of both blood vessels and cardiomyocytes [29, 30]. It was reported that acupuncture stimulation could not only change the adenosine concentration around the acupoints locally [15, 31] but also regulate the A2b expression in the cardiomyocytes [16]. The present study showed that in the EA group, the electroacupuncture pretreatment significantly enhanced A2b content and reduced the elevation of ECG ST segment, arrhythmias, and the myocardial infarct size. The enhancement of A2b and the cardioprotective effects were both blocked by the A2b-specific antagonist. In agreement with the previous study, the present results indicate that the cardioprotection produced by electroacupuncture pretreatment is mediated by A2b receptor [32].

Physiologically, calcium is known to be crucial for the systolic and diastolic activities of cardiomyocytes. The disorder of the intracellular calcium concentration like calcium overload is also one of the very important factors leading to pathological alterations of the cardiac cells [33, 34]. On the calcium signaling pathway of the cardiomyocytes RyR2, NCX, SERCA, and PLB are the key factors or Ca^2+^ signaling components which play a pivotal role in the balance of the intracellular Ca^2+^ concentration. For example, RyR2 is the major receptor on the membrane of the sarcoplasmic reticulum responsible for the release of Ca^2+^ from SR into the cytoplasm via a so-called calcium-induced calcium-release mechanism. Usually, a low heart rate or even a lethal arrhythmia will occur in the mice with an abnormal gene expression of RyR2 [35]. The present data showed that in the model group, the content of RyR2 which is a key SR membrane protein responsible for the Ca^2+^ release from the SR to cytoplasm increased significantly as compared with the NC group, suggesting that the RyR2 is involved in the cardiac injury described above, while a significant reduction of RyR2 in the EA group in comparison with that in the M group suggests that the SR membrane protein RyR2 participates in the mediation of the aforementioned cardioprotection produced by EA pretreatment.

NCX1 is one of the subtypes of NCX, an antiporter membrane protein mainly responsible for removing Ca^2+^ from the cytoplasm by exchanging Na^+^ into the cells, so as to participate the balancing of the intracellular Ca^2+^ concentration. Pathologically, NCX is reported to be associated with the delayed afterdepolarizations which may trigger cardiac arrhythmias [36]. Among the groups in the present study, NCX1 content was not changed significantly. However, a previous study shows that the elevated protein level of NCX1 is downregulated by electroacupuncture pretreatment [37]. The different results may be because the I/R model used in the present study is different from the abovementioned one. In the present study, the reperfusion time is only 15 min, much shorter than 80 min in the previous study mentioned above. Furthermore, NCX is known to be regulated by pH. Numerous pathological conditions are associated with drops in pH, which in turn affects NCX activity [38, 39]. Notably, I/R can reduce the intracellular pH, leading to an inhibitory influence on NCX transport that may contribute to the Ca^2+^ aberrations. Although the NCX1 content was not changed among the groups, the activity may be changed.

Another important membrane protein related to removal of Ca^2+^ from the cytoplasm is SERCA2a [40, 41]. During the diastolic period, most intracellular Ca^2+^ are taken back to the sarcoplasmic reticulum via SERCA2a, known to be prepared for triggering the next myocardial contraction [42]. The enhancement of Ca^2+^ uptake into the SR is considered to inhibit the removal of intracellular Ca^2+^ via NCX [43]. Impaired SERCA2a function is usually implicated in the abnormal diastolic or even systolic dysfunction [44]. As shown in the present results, the content of SERCA2a in the M group was markedly decreased as compared with that in the NC group, indicating that the reduced SERCA2a may at least partially mediate the cardiac dysfunction induced by I/R. In the EA group, the SERCA2a was increased obviously in comparison with the M group, indicating that EA pretreatment could elevate the protein level of SERCA2a in cardiomyocytes, which may contribute to the cardioprotection by enhancing the reuptake of intracellular Ca^2+^ into SR and decreasing the persistent contraction of cardiomyocytes during the overload of intracellular Ca^2+^. Actually, in the cardiomyocytes, SERCA is a predominant Ca^2+^ transporter because of the fact that it can remove more than 10 times intracellular Ca^2+^ as compared with NCX [45]. Taken together, the results suggest that on the I/R model used in the present study, SERCA2a, not NCX1, plays an important role in the mediation of cardioprotection produced by acupuncture pretreatment.

As an SR membrane protein with 52 amino acids, PLB usually inhibits the calcium-pumping function of SERCA in a reversible way [46, 47], mainly inhibiting the uptake of intracellular Ca^2+^ into the SR performed by SERCA2a. It is well known that heart failure is often accompanied with an impaired function in dealing with Ca^2+^ in the cardiomyocytes. Studies showed that in the early stage of heart failure, the reduction of phosphorylation of PLB accompanied with or without a decrease of SERCA2a expression led to an increase in the intracellular Ca^2+^ during diastole, which potentially results in a diastolic dysfunction [48, 49]. The present study showed that in the EA group, the P-PLB/PLB ratio was much higher than that in the M group. The P-PLB/PLB ratio is helpful for the uptake of intracellular Ca^2+^ into the SR, so as to possibly improve the impaired cardiac diastolic function.

Contraction of cardiac muscle is driven by an interaction between myosin and actin which is controlled by the transient binding of Ca^2+^ ions to Cardiac troponin C (cTn C) and activates thin muscle filaments on a beat-to-beat basis [50]. Cardiac troponin I (cTn I) inhibits the actomyosin ATPase on its own in a Ca^2+^-independent manner, and Ca^2+^ binding to regulatory sites located in the NH_2_-terminal domain of cTn C induces a conformational change that blocks the inhibitory action of cTn I and triggers muscle contraction [51, 52]. CTn I also plays a central role in ischemia-systolic dysfunction. Also, it is an inhibitory subunit of the troponin complex which acts as a Ca^2+^-dependent molecular switch by shuttling between actin (diastolic) and troponin C (systolic) [53]. When Ca^2+^ level is low, there is little interaction between cTn C and cTn I. It is a result that cTn I interacts with actin and inhibits it. When cTn C interacts with Ca^2+^, the interaction between cTn C and cTn I is enhanced to alleviate the interaction between cTn I and actin. Ca^2+^ binding to low-affinity sites in the N-terminal region of cTn C is the first step in the sequence of events associated with activation of myocardial contractile proteins [54]. CTn I regulates Ca^2+^ sensitivity on myofilaments by phosphorylation and intracellular environment and regulates myocardial contractility in a specific isomerism [55]. Calcium overload in cardiac myocytes is one of the important causes of myocardial ischemia injury [56]. Our previous experiments have also confirmed that electroacupuncture pretreatment can reduce the openness of L-type Ca^2+^ channels in cardiac myocytes that mimic whole-heart ischemia and ultimately alleviate myocardial injury caused by intracardiac calcium overload caused by ischemia [57]. The results show that cTn C/I ratio in the model group was significantly increased, which indicates that the cardiomyocytes were easier to bond with Ca^2+^, and the cardiomyocytes were prone to calcium overload injury, while the cTn C/I ratio in the EA group was significantly decreased, showing that the binding of cardiac myocytes to calcium was decreased and the occurrence of calcium overload after myocardial cell injury was reduced.

## 5. Conclusions

In conclusion, electroacupuncture pretreatment could reduce ischemia and reperfusion-induced myocardial injury via increasing possibly the A2b content and regulating the key Ca^2+^ signaling components, namely inhibiting RyR2 and enhancing P-PLB(S16)/PLB ratio and SERCA2a proteins, so as to diminish the intracellular Ca^2+^ overload and consequently lessen the myocardial injury.

## Figures and Tables

**Table 1:** Arrhythmia scoring system.

Arrhythmia scores	Type of arrhythmia
0	No arrhythmia
1	Atrial arrhythmias or an occasional PVC
2	Frequent PVC
3	VT (1∼2 episodes)
4	VT (>3 episodes) or VF (1-2 episodes)

PVC, premature ventricular contraction; VT, ventricular tachycardia; VF, ventricular fibrillation.

**Table 2:** The variation of proteins related to the calcium regulation.

Receptors/proteins	Roles played by the proteins	EA vs. M	EAG vs. EA
A2b	Related to calcium regulation	↑	↓
Ryanodine 2	Releasing Ca^2+^ from SR into the cytoplasm and related to the myocardial contraction	↓	↑
SERCA 2a	Calcium pump to uptake Ca^2+^ into SR and related to myocardial diastolic	↑	—
NCX1	Removing Ca^2+^ from the cytoplasm by exchanging Na^+^ into the cells	—	—
P-PLB(S16)/PLB	Regulating the function of SERCA2a, related to uptake intracellular Ca^2+^ into SR	↑	↓
Troponin C/I	The binding degree of cardiac myocytes to calcium	↓	↑

↑, upregulated; ↓, downregulated; —, unchanged.

## Data Availability

The data used to support the findings of this study are available from the corresponding author upon request.
